# Retinopathy of prematurity and placental histopathology findings: A retrospective cohort study

**DOI:** 10.3389/fped.2023.1099614

**Published:** 2023-02-23

**Authors:** Sam Ebenezer Athikarisamy, Geoffrey C. Lam, Matthew N. Cooper, Tobias Strunk

**Affiliations:** ^1^ Neonatal Directorate, Child and Adolescent Health Service, Perth, WA, Australia; ^2^ School of Medicine, University of Western Australia, Crawley, WA, Australia; ^3^ Department of Ophthalmology, Perth Children’s Hospital, Perth, WA, Australia; ^4^ Centre for Ophthalmology and Visual Science, University of Western Australia, Crawley, WA, Australia; ^5^ Wesfarmers' Centre for Vaccines and Infectious Diseases, Telethon Kids Institute, The University of Western Australia, Perth, WA, Australia

**Keywords:** retinopathy of prematurity, chorioamnionitis, funisitis, inflammation, oxygen

## Abstract

**Aim:**

Retinopathy of prematurity (ROP) is a biphasic vaso-proliferative disease that has the potential to cause blindness. In addition to prematurity and hyperoxia, perinatal infection and inflammation have been reported to play a critical role in the pathogenesis of ROP. The aim of this study was to assess the association between placental inflammation and the severity of ROP.

**Methods:**

A retrospective study of infants (<30 weeks of gestational age) born at the King Edward Memorial Hospital, a tertiary perinatal center in Western Australia.

**Results:**

A total of 878 infants were included in this study (ROP stage 0–2 = 829; 3 or more = 49). The presence of maternal chorioamnionitis appeared to show signs of an association with reduced odds of severe ROP: mild chorioamnionitis OR=0.43 (95% CI: 0.17, 1.05) and severe chorioamnionitis OR=0.68 (95% CI: 0.29, 1.60). A strong association was observed for oxygen supplementation at 36 weeks (OR: 5.16; *p* < 0.001), exposure to postnatal steroids (OR: 6.65; *p* < 0.001), and receipt of platelet transfusion (OR: 8.21; *p* < 0.001).

**Conclusion:**

Maternal chorioamnionitis or fetal chorioamnionitis was associated with reduced odds of severe ROP. A strong association was found in infants who needed oxygen supplementation at 36 weeks and those who required steroids or platelets in the postnatal period.

## Background

Retinopathy of prematurity (ROP) is a biphasic vaso-proliferative disease of the retina that almost exclusively affects preterm infants. Prematurity, in combination with relative hyperoxia after birth, leads to the arrest of physiological vascular development, involving obliteration and suppression of developing vessels (phase I). As the retina develops further, the metabolic demand exceeds supply, resulting in relative hypoxia and the excessive production of vascular growth factors and neovascularization (phase II) (1). Severe ROP can lead to poor visual outcomes, such as irreversible blindness, if not diagnosed and treated at the appropriate time.

Recent studies have shown that in addition to prematurity and hyperoxia, perinatal infection and inflammation may play a critical role in the pathogenesis of ROP (2). Acute inflammatory conditions of the placenta, such as chorioamnionitis (CA) (maternal host response), funisitis, and chorionic vasculitis (fetal inflammatory response), are caused by chemotactic gradients in the amniotic cavity. The accompanying fetal inflammatory response syndrome may increase the risk of ROP by directly sensitizing the developing retina to oxygen-induced changes in vascular endothelial growth factor (VEGF) levels. Animal studies suggest that perinatal infection and inflammation may sensitize the retina for the development of ROP (2).

The effects of inflammatory mediators and growth factors such as interleukin-1β can significantly increase the activity of hypoxia-inducible factor (HIF-1α) (3). In addition, there may be other inflammatory factors, such as phospholipase-2 and prostaglandins, that affect retinal neovascularization (4).

Clinical data on the association between placental histopathology and retinal outcomes in very preterm infants are conflicting (5, 6). This may partly reflect methodological heterogeneity, specifically the diagnostic criteria for chorioamnionitis (clinical vs. histological) and the gestational and postnatal ages of the study populations. The higher incidence of ROP in larger and more mature babies observed in low- and middle-income countries has been attributed to the increased incidence of infection in these cohorts (7).

In this study, we aimed to characterize the relationship between placental histology and ROP in a cohort of preterm infants < 30 weeks.

## Patients and methods

### Design and setting

This retrospective cohort study analyzed data from very preterm infants admitted to the only tertiary perinatal center in Western Australia.

### Participants

The electronic records of all infants born with a gestational age (GA) of 22 + 0 to 29 + 6 weeks delivered at or transferred to the study center were assessed for availability of placental histology and ROP disease classification. We excluded infants whose placentas were not available for histologic assessment.

### Data collection

Demographic characteristics and known risk factors for ROP, including sepsis, duration of oxygen requirement, duration of mechanical ventilation, and other common neonatal outcomes, were extracted from electronic databases where they were routinely recorded.

### Classification of placental histopathology

Histological examination of the placenta was performed as part of routine clinical care from pregnancies delivering at < 30 weeks’ GA. The findings were reported by a single senior placental histopathologist who was blinded to the clinical outcomes. Sections of the chorioamniotic membranes, umbilical cord, chorionic plate, and placenta were analyzed by one perinatal pathologist throughout the study period using an adaptation of a widely accepted semiquantitative scoring system. The presence and degree of maternal inflammation was defined by neutrophilic infiltration of the cellular chorion, of the membranes, or of the chorionic plate. The incidence and severity of fetal inflammation were defined by neutrophilic infiltration from the fetal vessels into the umbilical vessels or the chorionic plate vessels. Funisitis was defined as inflammation of the umbilical cord arising from the fetal vessels (8).

### ROP screening and treatment protocol

All neonates born < 31 weeks postmenstrual age, regardless of birth weight, and all neonates with birth weight < 1,250 grams, regardless of postmenstrual age at birth, were screened for ROP as part of routine care.

The screening examination for ROP followed the guidelines proposed by the American Academy of Ophthalmology and Pediatrics and the Association for Pediatric Ophthalmology and Strabismus (9). The first screening examination was performed at 30**–**31 weeks postmenstrual age in infants born at < 27 weeks GA and at 4**–**5 weeks postnatal age in infants born at 27**–**32 weeks GA. After the first evaluation, if the infant did not have ROP, the neonate was evaluated at 2–4-week intervals until full vascularization. If the patient had an active or rapid progressive lesion, evaluation was more frequent, depending on the clinical findings.

ROP status was recorded for each infant on the basis of the International Committee for the Classification of Retinopathy of Prematurity (ICROP) guidelines (10, 11). In this study, the findings were recorded using the ICROP 2 guidelines, as the study cohort predated the 2021 update to ICROP 3. From this cohort, three groups were defined: “no ROP,” “mild ROP” (for stage 1 or 2), and “severe ROP” (for stage 3 or 4; no stage 5 cases were observed). ROP was treated primarily by laser surgery, and treatment decisions were based on the ET-ROP study guidelines (12). Laser was performed with advanced ROP, particularly stage III with plus disease, significant preplus, or “Aggressive Posterior ROP” (APROP; now called “aggressive ROP”) after review by the ophthalmologist. Anti-VEGF treatment was reserved for infants who had Zone 1 disease and for infants who were too unstable to undergo laser surgery.

### Statistical analysis

Following the extraction of data from medical records, the data were structured in the form of one record (row) per child. Descriptive statistics (mean and standard deviation; count and percentage) were calculated for the extracted demographic and clinical variables and are presented by ROP classification. Bivariate assessments of the associations between clinical variables and ROP classification were carried out using a chi-squared test followed by ordinal regression [where odds ratios and 95% confidence intervals (OR; 95% CI) are reported]; the Student's t-test was used for comparison of continuous (normally distributed) data between groups. Further assessment of the association between clinical risk factors and the incidence of severe ROP was carried out using logistic regression. Given the limited size of the dataset, only a minimal set of known confounders were adjusted for in the model (mean-centered gestational age, mean-centered birth weight, and sex), while the placental histopathology variables were entered into the model in turn and then together; ORs with 95% CIs are reported. Post-hoc analysis of a combined maternal and fetal variable with three levels (neither; either maternal or fetal but not both; or both) was carried out in an effort to further understand the relationship between these variables and ROP. All analyses were conducted using R (13).

## Results

**Figure 1:**
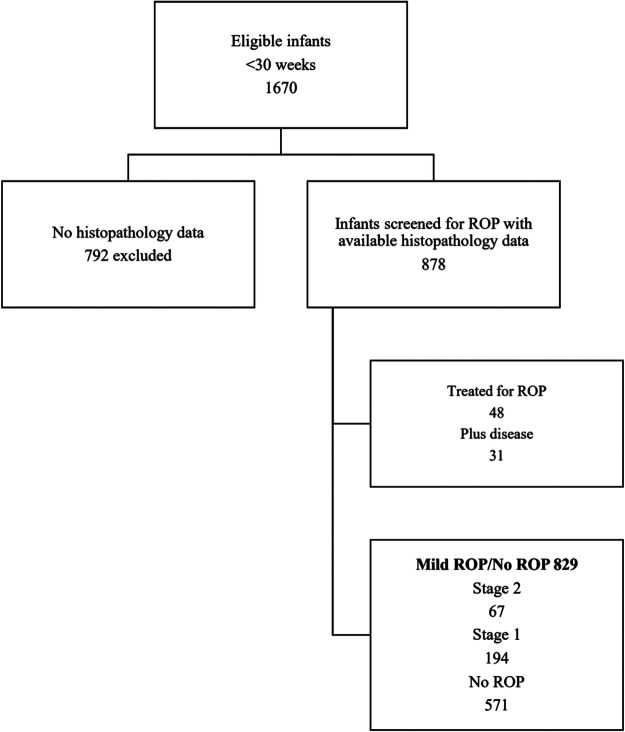
Flow of included infants according to stage of ROP.

Among newborns identified as eligible during the study period (n = 1,670 infants), placental histology and ROP outcome data were available for 878 infants. There were 49 infants with stage 3 or more severe disease and 829 infants with milder disease (stage 0**–**2). Forty-eight infants needed treatment for ROP, in 31 of whom signs of plus disease were present. Consistent with known risk factors, infants with plus disease were smaller (mean birth weight 669 g vs. 993 g; *p* < 0.001) than those without. Patient flow and distribution according to ROP stage are depicted in Figure 1.

Descriptive statistics for demographic and clinical variables, by ROP classification, are given in Table 1. The distribution of retinopathy of prematurity classified by histological characteristics is shown in Table 2. A strong association with increasing severity of ROP was observed for oxygen supplementation at 36 weeks (OR: 5.16; 95% CI: 3.81,7.00; *p* < 0.001), exposure to postnatal steroids (OR: 6.65; 95% CI: 4.02, 11.01; *p* < 0.001), and receipt of platelet transfusion (OR: 8.21; 95% CI: 4.13,16.34; *p* < 0.001). In our cohort, more mature gestational age (OR: 0.65; 95% CI: 0.51,1.85; *p* < 0.01; per 1-week increase) and higher birth weight (OR: 0.62; 95% CI: 0.49,0.79; *p* < 0.001; per 100 g increase) were associated with decreased odds of severe ROP, and male sex was associated with increased odds (OR: 2.06; 95% CI: 1.08, 3.96; *p* < 0.05) of severe ROP based on a logistic regression model (with only these three variables included).

**Table 1:** Demographic and clinical characteristics.

		No ROP (stage 0)	Mild ROP (stages 1 and 2)	Severe ROP (stage 3 or higher)
Inborn		531 (93.3%)	250 (96.2%)	47 (95.9%)
IUGR		74 (13.0%)	38 (14.6%)	11 (22.4%)
Multiple birth		149 (26.2%)	64 (24.6%)	8 (16.3%)
Sex	Male	324 (56.9%)	123 (47.3%)	32 (65.3%)
	Female	245 (43.1%)	137 (52.7%)	17 (34.7%)
Birth weight	<500	2 (0.4%)	11 (4.2%)	4 (8.2%)
	501–750	57 (10.0%)	109 (41.9%)	34 (69.4%)
	751–1000	176 (30.9%)	96 (36.9%)	9 (18.4%)
	>1,000 grams	334 (58.7%)	44 (16.9%)	2 (4.1%)
Gestational age	23–25^+6^	57 (10.0%)	135 (51.9%)	39 (79.6)
	26–28^+6^	316 (55.5%)	116 (44.6%)	9 (18.4%)
	29–30	196 (34.4%)	9 (3.5%)	1 (2.0%)
Antenatal steroids complete	None	41 (7.2%)	13 (5.0%)	2 (4.1%)
	<24 h	139 (24.4%)	62 (23.8%)	13 (26.5%)
	>24 h	227 (39.9%)	127 (48.8%)	26 (53.1%)
	>7 days	162 (28.5%)	58 (22.3%)	8 (16.3%)
Mode of delivery	Vaginal	216 (38.0%)	114 (43.8%)	21 (42.9%)
	CS	353 (62.0%)	146 (56.2%)	28 (57.1%)
Apgar < 7 at 1 min		385 (67.7%)	213 (81.9%)	45 (91.8%)
Apgar < 7 at 5 min		132 (23.2%)	76 (29.2%)	19 (38.8%)
Platelet transfusion		4 (0.7%)	19 (7.3%)	7 (14.3%)
Maternal antibiotics		303 (53.3%)	163 (62.7%)	32 (65.3%)
Postnatal steroids		15 (2.6%)	35 (13.5%)	14 (28.6%)
Oxygen at 36 weeks		97 (17.0%)	123 (47.3%)	34 (69.4%)
Nitric oxide		8 (1.4%)	7 (2.7%)	4 (8.2%)
Early-onset sepsis		8 (1.4%)	7 (2.7%)	4 (8.2%)
Late-onset sepsis		104 (18.3%)	50 (19.2%)	8 (16.3%)
NEC		17 (3.0%)	8 (3.1%)	4 (8.2%)
IVH	No IVH	9 (1.6%)	3 (1.2%)	0 (0.0%)
	Grade 1	104 (18.3%)	50 (19.2%)	8 (16.3%)
	Grade 2	31 (5.4%)	18 (6.9%)	10 (20.4%)
	Grade 3	8 (1.4%)	6 (2.3%)	0 (0.0%)
	Grade 4	18 (3.2%)	15 (5.8%)	2 (4.1%)
	Not recorded	399 (70.1%)	168 (64.6%)	29 (59.2%)

ROP: retinopathy of prematurity; NEC: necrotizing enterocolitis.

**Table 2:** Distribution of retinopathy of prematurity classified by histological characteristics.

		No ROP (stage 0)	Mild ROP (stage 1 and 2)	Severe ROP (stage 3 or higher)
Maternal chorioamnionitis	No	302 (53.1%)	110 (42.3%)	23 (46.9%)
	Mild focal	164 (28.8%)	74 (28.5%)	10 (20.4%)
	Severe	82 (14.4%)	65 (25.0%)	15 (30.6%)
	Not specified	21 (3.7%)	11 (4.2%)	1 (2.0%)
Fetal chorioamnionitis	No	369 (64.9%)	137 (52.7%)	29 (59.2%)
	In cord	10 (1.8%)	7 (2.7%)	2 (4.1%)
	In chorionic plate	99 (17.4%)	60 (23.1%)	6 12.2%)
	In cord and chorionic plate	91 (16.0%)	56 (21.5%)	12 (24.5%)
Placental ischemia	No	467 (82.5%)	226 (87.6%)	38 (77.6%)
	Yes	101 (17.8%)	56 (21.5%)	12 (24.5%)
Placental organisms isolated	No	0 (0.0%)	1 (0.4%)	1 (2.0%)
	1 organism	554 (97.4%)	250 (96.2%)	46 (93.9%)
	2 or more	15 (2.6%)	9 (3.5%)	2 (4.1%)
Funisitis	No	468 (82.5%)	204 (78.5%)	37 (75.5%)
	Present	101 (17.8%)	56 (21.5%)	12 (24.5%)

After controlling for GA, birth weight, and sex, the presence of maternal chorioamnionitis appeared to show signs of an association with reduced odds of severe ROP: mild chorioamnionitis OR=0.43 (95% CI: 0.17,1.05) and severe chorioamnionitis OR=0.68 (95% CI: 0.29,1.60). While the confidence intervals included 1, the ORs were relatively large in magnitude and somewhat consistent across the levels of maternal chorioamnionitis. After controlling for GA, birth weight, and sex, the presence of fetal chorioamnionitis in the “chorionic plate” appeared to show a significant association with reduced odds (OR: 0.29; 95% CI: 0.11, 0.82) of severe ROP.

**Figure 2:**
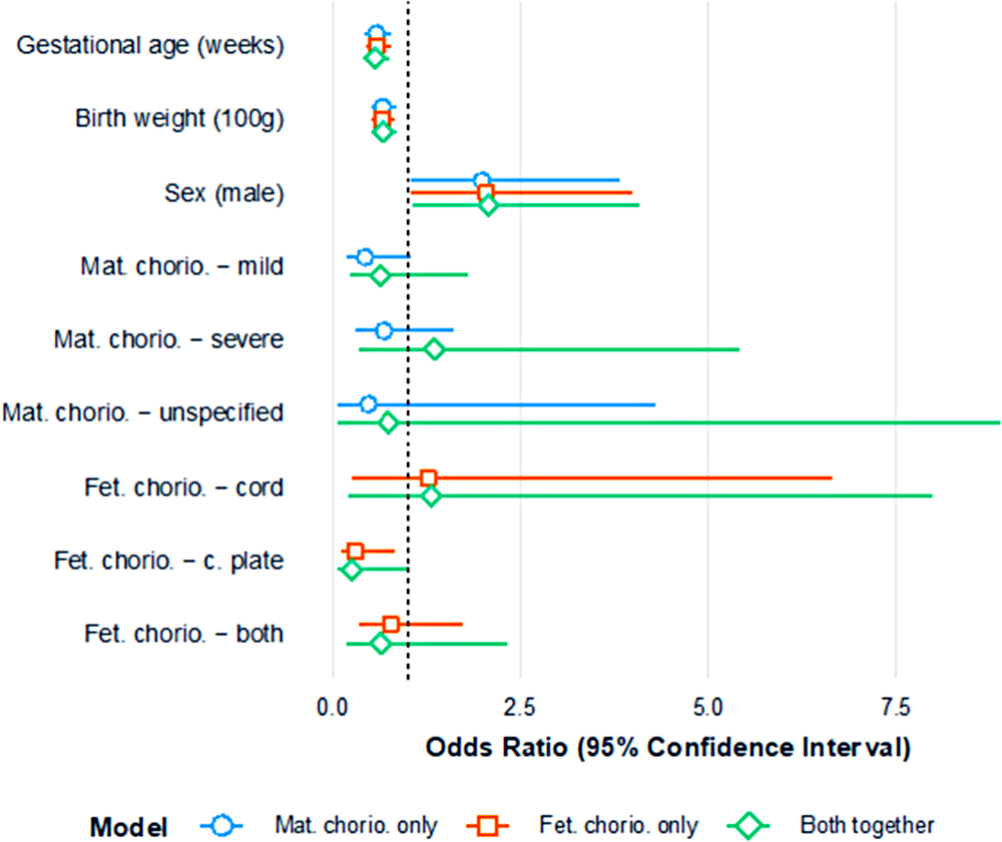
Plot of odds ratios (and 95% confidence intervals) for severe ROP; three models assessing maternal chorioamnionitis (minimally adjusted) and fetal chorioamnionitis (minimally adjusted) separately; both maternal chorioamnionitis and fetal chorioamnionitis modeled together.

When the effects of maternal and fetal chorioamnionitis were examined in the model together (Figure 2), there was some attenuation of the effect. For the most part, the results were stable, with the exception of the OR for maternal severe chorioamnionitis, which became 1.35 (95% CI: 0.34, 5.42); the reduced odds of severe ROP when fetal chorioamnionitis in the “chorionic plate” persisted (OR: 0.26; 95% CI: 0.07, 1.00). The *post-hoc* combined variable for maternal or fetal chorioamnionitis suggested a reduced risk of severe ROP with just one type of chorioamnionitis (OR: 0.56; 95% CI: 0.21, 1.55) or both maternal and fetal chorioamnionitis together (OR: 0.48; 95% CI: 0.22, 1.07), acknowledging that both confidence intervals included 1. The addition of placental organisms isolated to the model suggested that this variable had little association with severe ROP (OR: 0.91; 95% CI: 0.18, 2.32). The addition of funisitis to the model also suggested that this had little association with severe ROP (OR: 0.92; 95% CI: 0.32, 2.62).

## Discussion

The relationship between placental inflammation and risk for ROP has been studied in both clinical and experimental setups. A systematic review by Mitra et al. did not show any association between CA and ROP (any stage) or with severe ROP (stage 3 or higher) (5). The authors concluded that CA cannot be definitively considered a risk factor for ROP, and further studies should adjust for potential confounding factors and report results by stage to clarify the association with severe ROP. This retrospective study aimed to determine whether there was an association between different stages of histological inflammation and the severity of ROP and to examine common confounding factors. The most recent systematic review by Villamor et al. (2019) stated that histological CA is a risk factor for developing ROP. The review observed a significant positive association between any CA and all stages of ROP. This association was significant for histological but not for clinical CA (6).

In our study, after controlling for GA, birth weight, and sex, the presence of both mild and severe maternal chorioamnionitis appeared to show signs of an association with reduced odds of severe ROP. The OR was 0.43 (95% CI: 0.17, 1.05) for mild chorioamnionitis and 0.68 (95% CI: 0.29, 1.60) for severe chorioamnionitis. Owen et al. recently reported that acute placental inflammation, analyzed by severity, revealed significant inverse associations for both maternal-level (*p* = 0.01) and infant-level (*p* = 0.005) inflammation with the presence of ROP disease (14). Park et al. reported, on the basis of a multiple logistic regression analysis (of 85 infants), that amnionitis (OR: 0.120, *p* = 0.014) and inflammation in Wharton's jelly (odds ratio: 0.124, *p* = 0.018) were independent protective factors against ROP. It is postulated that with the progression of acute histologic chorioamnionitis, there is a decrease in the level of the intact form of insulin-like growth factor binding protein 1, ultimately affecting IGF-1 bioavailability (15, 16).

A study by Ahn et al. found that the incidence of chorioamnionitis was higher in the APROP infants compared to the infants without APROP (*p* < 0.001) (17). This is significant, given the higher rate of complications and unfavorable anatomical outcomes following APROP. In our cohort, we could not identify the exact number of APROP cases because of the retrospective nature of the study. However, 14 infants received treatment at a milder stage, which could have been due to the presence of APROP. Kim et al. found a significant association between chronic placental inflammation and severe ROP requiring treatment (adjusted odds ratio: 2.739; *p* = 0.029) (18).

In a retrospective cohort of 246 infants, a multivariate regression model showed that elevated maternal WBC was associated with ROP progression needing laser treatment (19). The authors could not find any association with histologic chorioamnionitis. A similar finding was noted in our cohort, where maternal or fetal CA did not increase the risk; in contrast, it reduced the risk. In another study involving a retrospective cohort that adjusted for covariates (*n* = 1217), both maternal and fetal inflammatory markers together posed a significant risk for severe ROP (*p* = 0.03) (20).

In our cohort of infants, neither early- nor late-onset sepsis had a significant effect on incidence of ROP. The numbers were too small to examine whether sepsis was associated with a higher incidence of plus disease. Wang et al., in a systematic review that had large heterogeneity, found that sepsis increased the risk of ROP in preterm infants (21). Al-Essa et al. found that in preterm infants, sepsis increased the risk of ROP 3.5-fold (22). A higher risk (OR = 6.86) was found by Araz et al. for the development of severe ROP (23).

In this study, the results of the addition of “presence of placental organism” to the model suggests that this variable is not associated with severe ROP (OR: 0.91; 95% CI: 0.18, 2.32). The authors of the ELGAN study (715 infants; GA < 27 weeks) reported that the co-occurrence of bacteria in the placenta and inflammation increased the risk of ROP in Zone 1. If either of these presented alone, it did not increase the risk (24). In our retrospective cohort, information on zone was not available; hence, this observation could not be validated. There was an increasing severity of ROP seen in infants needing postnatal platelet transfusion (OR: 8.21; 95% CI: 4.13, 16.34; *p* < 0.001) in our cohort. A recent review that included 19 studies concluded that there was an association between platelet deficiency and severe ROP, but this review could not estimate a critical threshold (25).

Our cohort showed a strong association with increasing severity of ROP in infants who needed postnatal steroids (OR: 6.65; 95% CI: 4.02, 11.01; *p* < 0.001). A similar piece of research involving a retrospective study of 75 infants using a multivariate logistic regression identified “total dosage of systemic steroids” as a risk factor predicting treatment warranting ROP. On receiver operating characteristic (ROC) curve analysis, a cut-off value of 8.95 mg/kg steroid was found to be significant (26). We could not calculate the cumulative dose of steroids in our infants.

Current treatment strategies, both laser and anti-VEGF agents, have their own long-term effects on visual and long-term neurological outcomes. Although anti-VEGF agents have the advantage of being less destructive to the retina, their long-term effects on the developing brain are still being studied. A large body of evidence has emerged to date from the oxygen-induced retinopathy (OIR) models and its molecular pathogenesis, and this does not represent the patient cohort that we see in the clinical world (27). We need more epidemiological data that capture all information, including what happens early in pregnancy and the intrapartum period.

A major strength of this study is the large sample size and the fact that the data included detailed histological assessment of the placenta, including microbiological data. One of the key limitations of this study is its retrospective nature and the exclusion of infants due to a lack of placental histopathology findings. Zone-specific details could have provided more information about the effect of chorioamnionitis on the developing retina.

## Conclusions

After controlling for GA, birth weight, and sex, the presence of maternal chorioamnionitis or fetal chorioamnionitis on its own appears to show signs of an association with reduced odds of severe ROP. When the effects of maternal and fetal chorioamnionitis were examined in the model together, there was some attenuation of the effect. The results of the addition of isolated placental organisms or funisitis to the model suggest that these variables are not associated with severe ROP. As reported in the literature, a strong association with increasing severity of ROP was observed in infants who needed oxygen supplementation at 36 weeks and who needed steroids and platelet transfusion in the postnatal period.

## Data Availability

The original contributions presented in the study are included in the article/supplementary material, further inquiries can be directed to the corresponding author.
